# *t*-Test at the Probe Level: An Alternative Method to Identify Statistically Significant Genes for Microarray Data

**DOI:** 10.3390/microarrays3040340

**Published:** 2014-12-16

**Authors:** Marcelo Boareto, Nestor Caticha

**Affiliations:** Institute of Physics, University of São Paulo, São Paulo, SP 05508-900, Brazil; E-Mail: nestor@if.usp.br

**Keywords:** microarrays, preprocessing, variance shrinkage, *t*-test, background correction

## Abstract

Microarray data analysis typically consists in identifying a list of differentially expressed genes (DEG), *i.e.*, the genes that are differentially expressed between two experimental conditions. Variance shrinkage methods have been considered a better choice than the standard *t*-test for selecting the DEG because they correct the dependence of the error with the expression level. This dependence is mainly caused by errors in background correction, which more severely affects genes with low expression values. Here, we propose a new method for identifying the DEG that overcomes this issue and does not require background correction or variance shrinkage. Unlike current methods, our methodology is easy to understand and implement. It consists of applying the standard *t*-test directly on the normalized intensity data, which is possible because the probe intensity is proportional to the gene expression level and because the *t*-test is scale- and location-invariant. This methodology considerably improves the sensitivity and robustness of the list of DEG when compared with the *t*-test applied to preprocessed data and to the most widely used shrinkage methods, Significance Analysis of Microarrays (SAM) and Linear Models for Microarray Data (LIMMA). Our approach is useful especially when the genes of interest have small differences in expression and therefore get ignored by standard variance shrinkage methods.

## 1. Introduction

Microarrays are a widely used methodology for measuring the expression of thousands of genes simultaneously. A common application of microarrays is to compare the expression levels of genes in samples drawn from two different experimental conditions in order to determine which genes are differentially expressed. Typically, a microarray dataset has in the order of ten thousand genes, whereas only a small subset of these genes is relevant, and the number of samples range from a few tens up to a few hundreds. Multiple processing steps are required in order to identify the genes of interest and errors in these steps compromise the reliability of the analysis. As a result, the comparison of the lists of differentially expressed genes (DEG) reported by different groups have revealed very small overlap [1,2]. Therefore, understanding the sources of this lack of robustness and the development of more reliable methods for the identification of the DEG remains a crucial issue.

The preprocessing of the raw probe intensity data constitutes the initial step in microarray data analysis and its goal is to infer a variable that represents the gene concentration. Usually, the preprocessing analysis is performed in three steps: normalization, background correction and summarization. First, the normalization is performed to reduce sources of variation of non-biological origin among the arrays in order to make them comparable. Next, in background correction, the background intensity due to non-specific hybridization and optical noise is inferred and subtracted from the normalized intensity. Finally, in the summarization step, the multiple probe intensities for each probe set is combined into a single gene expression value.

The probe intensity measure *Y* can be modeled as a combination of background *B* (due to optical noise and non-specific hybridization) and specific hybridization *S* [3,4]:

(1) $$Y_{ijg}=B_{jg}+S_{ijg}=B_{jg}+\phi_{jg}\theta_{ig}$$

where $\theta_{ig}$ denotes the expression measure for the gene *g* in the *i*th sample, and the indexes *i*, *j* and *g* represent the sample, the probe and the gene, respectively. This model assumes that the intensity value $Y_{ijg}$ increases linearly as $\theta_{ig}$ increases, but the rate of increase of the expression $\theta_{ig}$ is different for each probe *j* and is represented by $\phi_{jg}$. It is also assumed that after normalization, the background $B_{jg}$ is independent of the sample [5].

Background correction is the most critical step because errors associated with the inference of this variable strongly affect the genes with low expression values—therefore decreasing the statistical power of further analysis. In the Affymetrix platform it is common to include an extra probe—referred to as the mismatch—created by changing the middle (13th) base with the intention of directly measuring the effect of background noise. The methods dChip [3] as well as the Affymetrix methods MAS5.0 and PLIER [6] use the mismatch intensity as an estimate of the background. Therefore, Irizarry *et al.* [4] showed that subtracting the mismatch intensity from the perfect match intensity (*Y*) results in expression estimates with an exaggerated error, mainly for low expression values. Thus, they proposed a background adjustment step that ignores the mismatch intensities, named RMA [4], which uses the posterior mean E[S|Y] as a background adjustment.

Once a gene expression estimation is obtained, it is common to take the logarithmic transformation of this variable because the difference in transformed data, the fold change, is considered easier to manipulate and interpret. Some preprocessing algorithms return the expression concentration in log scale (like RMA and Plier) while others do not (like MAS5.0). It seems that there is no consensus about what scale should be chosen when using *t*-test or others ranking methods. Nevertheless, this choice is important because the *t*-test, for example, is not invariant under monotone transformations, *i.e.*, the results of the test is different if *x* is replaced by $\log_{2}x$.

After preprocessing, several types of statistical tests can be applied in order to find the differentially expressed genes under two conditions. The independent *t*-test is the most popular statistical approach to select differentially expressed genes presumably due to its simplicity to implement and interpret. In this test, it is assumed that the data follows a normal distribution and, under the null hypothesis $H_{0}$, the average of the gene expression in both experimental conditions are the same. A discordance of the data from what is specified in $H_{0}$ can be quantified as the probability of observing a value for the test statistic that is at least as extreme as the value that was actually observed. This probability *p* is referred to as the *p*-value and a threshold value *α* can be chosen so that the hypothesis $H_{0}$ is rejected if p≤α. Then, a statistical significance can be assigned, which is a statistical assessment of whether observations reflect a pattern rather than just chance. The *t* variable, which follows a Student’s t-distribution when the normality of the data holds, is defined as:

(2) $$t_{g}=\frac{{\langle {\hat{\theta }}_{gi}\rangle }_{i\in A}-{\langle {\hat{\theta }}_{gi}\rangle }_{i\in B}}{s_{g}}$$

where ${\hat{\theta }}_{ig}$ is the inferred expression level by a preprocessing method and ${\langle {\hat{\theta }}_{gi}\rangle }_{i\in A}$ and ${\langle {\hat{\theta }}_{gi}\rangle }_{i\in B}$ are the expression averages over the samples *i* under the conditions *A* and *B*, respectively.

The empirical standard deviation is defined as

(3) $$s_{g}=\sqrt{a\left(\sum_{i\in A}{\left[{\hat{\theta }}_{gi}-{\langle {\hat{\theta }}_{gi}\rangle }_{i\in A}\right]}^{2}+\sum_{i\in B}{\left[{\hat{\theta }}_{gi}-{\langle {\hat{\theta }}_{gi}\rangle }_{i\in B}\right]}^{2}\right)}$$

where $a=\left(1/n_{A}+1/n_{B}\right)/\left(n_{A}+n_{B}-2\right)$ and the constants $n_{A}$ and $n_{B}$ represent the number of samples under the experimental conditions *A* and *B*, respectively.

Despite being widely used, the *t*-test has been subject to criticism in the literature since the error in preprocessed data tend to be asymmetric [7,8,9,10]. As a consequence, the variance estimation is dependent on the expression level because the genes with low expression levels are more affected by the errors in background correction. Because of that, modified versions of the standard *t*-test have been developed as alternative approaches [11,12,13,14,15]. Those approaches modify the *t*-test by using a procedure called variance shrinkage, which consists in modifying the denominator of the *t* variable by combining the gene-specific variance and a predictive variance.

The method Significance Analysis of Microarrays (SAM) [11] is the most popular alternative to *t*-test and it consists in a modification of the standard *t*-test by the inclusion of an extra variable $s_{0}$, added to the pooled variance $s_{g}$. The extra variable $s_{0}$ is chosen in order to minimize the dependency of *t* on the expression level ${\hat{\theta }}_{g}$ and the significant genes are identified comparing the modified *t* variable with a similar variable obtained under random permutations among the samples. Modifications of the *t*-test based on Empirical Bayes [13,14,16] have also been widely used and have been considered a good choice [9], and the method Linear Models for Microarray Data (LIMMA) [14] is the most widely used.

There is no shortage of more sophisticated alternatives to the *t*-test. However, given the widespread tendency to use the standard *t*-test, understanding the reason of its poor performance remains a crucial issue. Here, we show that the standard *t*-test can be used at the probe level, skipping background correction by using the normalized intensity data directly in the *t*-test. Several methods have approached this problem at the probe level [17,18,19,20], but all these approaches use background-corrected data. Our methodology outperforms the standard *t*-test using preprocessed data and the most used shrinkage methods SAM and LIMMA, therefore suggesting that background correction is a major source of error in microarray analysis. The methods were compared in terms of sensitivity by using the Affymetrix spike-in dataset, a commonly used benchmark, and in terms of robustness by using leukemia, breast cancer and multiple myeloma datasets.

## 2. Methodology

The standard *t*-test is scale- and location-invariant, *i.e.*, the results of the test do not change if *x* is replaced by ax+b, where *a* and *b* are constants. Because of that, according to the model described in Equation (1), applying the *t*-test to the normalized intensity data is equivalent to applying the test on the concentration variable *θ*, as illustrated in the following manipulations:

$$\begin{array}{cccc} t_{jg} & = & \frac{{\langle Y_{ijg}\rangle }_{i\in A}-{\langle Y_{ijg}\rangle }_{i\in B}}{s(Y_{ijg})}\\ & = & \frac{{\langle B_{jg}\rangle }_{i\in A}+\phi_{jg}{\langle \theta_{ig}\rangle }_{i\in A}-{\langle B_{jg}\rangle }_{i\in B}-\phi_{jg}{\langle \theta_{ig}\rangle }_{i\in B}}{\phi_{jg}s\left(\theta_{ig}\right)}\\ & = & \frac{\phi_{jg}\left[{\langle \theta_{ig}\rangle }_{i\in A}-{\langle \theta_{ig}\rangle }_{i\in B}\right]}{\phi_{jg}s\left(\theta_{ig}\right)}\\ & = & {\left[\frac{{\langle \theta_{ig}\rangle }_{i\in A}-{\langle \theta_{ig}\rangle }_{i\in B}}{s(\theta_{ig})}\right]}_{j} & (4) \end{array}$$

Note that no log transform was performed in the data and that for each gene *g* a set of *t* variables (one for each probe) is obtained. In order to select the differentially expressed genes, we propose to take the median *t*-values, since the median is more robust than the average in the presence of outliers. Then, the genes can be ranked according to its relevance by the median *t*-values, which often is enough for selecting a subset of genes as biomarkers candidates.

To estimate the statistical significance of the median *t*-values of each gene, we define F(t) and f(t) to be respectively the cdf and pdf distribution of a *t*-variable. Then, for the case of a number of probes equal to *n*, the pdf distribution of the median *t*-value g(t) is given by [21]:

(5) $$g\left(t\right)=\left\{\begin{array}{cc} C_{n}{\left[F\left(t\right)\right]}^{n/2}{\left[1-F\left(t\right)\right]}^{n/2}f\left(t\right) & \;\text{if}\;n\;\text{is}\;\text{even}\;\text{and}\;C_{n}=\frac{n!}{(n/2)!(n/2)!}\\ C_{n}{\left[F\left(t\right)\right]}^{(n-1)/2}{\left[1-F\left(t\right)\right]}^{(n-1)/2}f\left(t\right) & \;\text{if}\;n\;\text{is}\;\text{odd}\;\text{and}\;C_{n}=\frac{n!}{(n-1)/2!(n-1)/2!} \end{array}\right.$$

Now, for a given *t*-median value $t_{m}$, a *p*-value can be estimated by integrating the pdf g(t) function for $t<t_{m}$.

## 3. Results

### 3.1. Sensitivity

The sensitivity in identifying the differentially expressed genes was evaluated by using spike-in experiments. This dataset was obtained from measurements on specifically constructed and controlled DNA microarrays experiments using human genome HG-U133. These experiments were designed by Affymetrix for the purpose of developing and validating the Affymetrix Microarray Suite (MAS) 5.0 expression algorithm [22]. The samples follow a Latin Square design consisting of 42 genes in 14 different concentrations (with 3 repetition each), see Table A1 in the Appendix.

To simulate an analysis in which two conditions are compared, we rearranged the data as follows. First, the genes from samples 1 to 3 were attributed to condition *A* and those from samples 4 to 6 were attributed to condition *B*. The exceptions were the genes from samples 40 to 42 because we want to probe the effect of small differences in expression, *i.e.*, we only evaluate differences in the fold change equal to 2. We kept the procedure using the replications 4–6 as condition *A* and 7–9 as condition *B*, again excluding the 0 and 512 pM differences. We ended up with 14 “experiments” in the two conditions and 23,000 genes of which 39 are differentially expressed. This procedure is similar to the one done in Affycomp [23] to obtain a Receiver Operating Characteristic (ROC) curve with fold change equal to 2.

**Figure 1:**
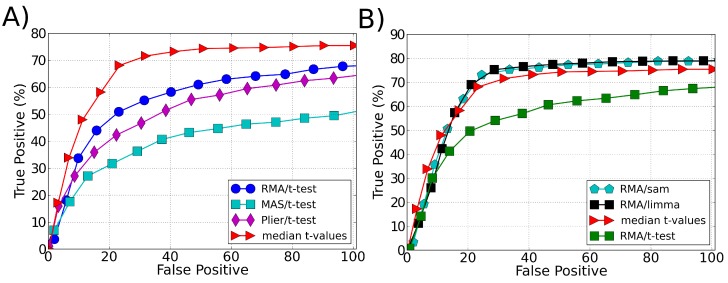
ROC curve comparing the performance (sensitivity) of different ranking methodologies in identifying differentially expressed genes in spike-in data. (**A**) Comparison of our approach (median *t*-values) with different preprocessing methods used with *t*-test as ranking method to select the differentially expressed genes. (**B**) Comparison of our approach (median *t*-values) with the best performance of other ranking methods: *t*-test, SAM and LIMMA. The best performance of *t*-test, SAM and LIMMA is obtained when using RMA as a preprocessing algorithm.

The results are presented as an ROC curve whose definition is given by a true positive (TP) rate against a false positive (FP) rate obtained at different threshold values. To plot a single average ROC curve, we calculated the average TP and FP over the 14 experiments obtained in the Affymetrix spike-in data rearrangement. The proposed approach showed a significant improvement on the sensitivity in recognizing the differentially expressed genes when the *t*-test was used as the ranking method, Figure 1A. Its performance is similar to the SAM and LIMMA best performance (when using RMA as a preprocessing algorithm), Figure 1B. In all applied tests the data were normalized using the quantile normalization method [24].

### 3.2. Robustness

In addition to having a good sensitivity, a good method for selecting the differentially expressed genes (DEG) should be robust, *i.e.*, the lists of DEG generated by different samples should share a good fraction of genes. The lack of agreement between those lists is a well-known issue in microarray analysis, mainly in cancer studies [1,2]. Furthermore, a good reliability of the selected DEG correlates positively with the class predictability [25]. In order to assess the robustness of a method, we used a natural similarity measure introduced by Ein-Dor *et al.* [2], which is the fraction of genes shared by two lists of DEG obtained from different samples using a given method. More specifically, in order to estimate the robustness of the methods, we generated 100 training samples by taking a subset of *n* experiments chosen randomly. For each training sample we chose the $N_{top}=100$ most significant genes obtained by the given method. Then, we compared the fraction of shared genes ($f_{a,b}$) between the training samples *a* and *b*. The average of the fraction of shared genes over all combinations of two training samples ($f={\langle f_{a,b}\rangle }_{a\neq b}$) is the figure of merit with which we represent and evaluate the robustness. Therefore, the closer is *f* to 1, the more robust is the method.

We calculated the average overlap for leukemia, breast cancer and multiple myeloma datasets for different sample sizes. The leukemia dataset consists of 24 samples of acute lymphoblastic leukemia (ALL) patients, 28 samples of acute myelogenous leukemia (AML) patients and 20 samples of mixed-lineage leukemia (MLL) patients [26]. We chose only the samples of leukemia type ALL and AML because these two types can be clearly distinguished based solely on gene expression profiles [27]. The Breast Cancer and Multiple Myeloma datasets were obtained from the MicroArray Quality Control (MAQC) consortium [25]. The Breast Cancer dataset can be divided according to two endpoints, pre-operative treatment response (pCR, pathologic complete response) and estrogen receptor (ER). The Multiple Myeloma dataset can be divided according to overall survival milestone outcome (OS-MO) and event free survival milestone outcome (EFS-MO), see Table A2 in the Appendix.

**Figure 2:**
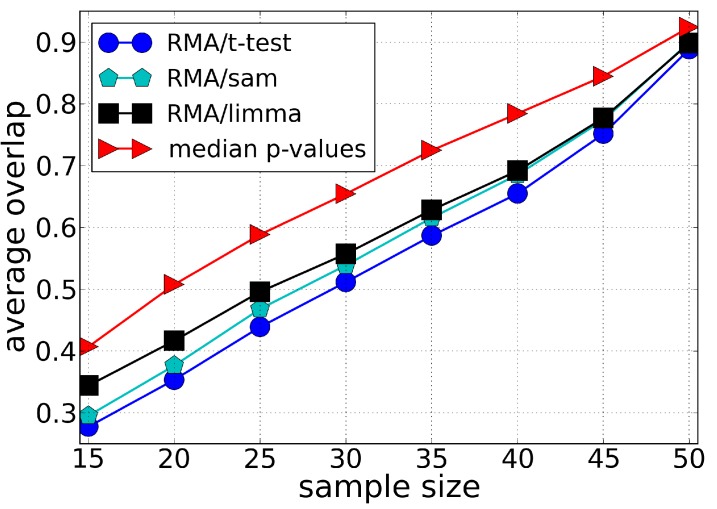
Average of the fraction of genes shared by two lists of differentially expressed genes (overlap) as a function of the sample size using the Leukemia dataset. Each list of differentially expressed genes is composed by the top 100 genes chosen according to different ranking methods, *i.e.*, *t*-test, SAM and LIMMA (preprocessed by the RMA method), and our approach (median *t*-value) which does not require a preprocessing algorithm. The average value of the overlap between the lists is calculated over 100 lists chosen randomly.

**Figure 3:**
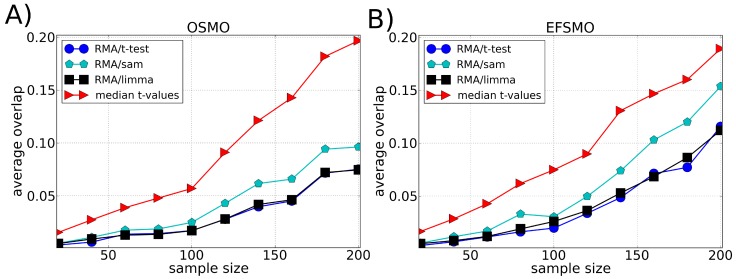
Average of the fraction of genes shared by two lists of differentially expressed genes (overlap) as a function of the sample size using the Multiple Myeloma dataset divided according to (**A**) Overall Survival Milestone Outcome (OS-MO) and (**B**) Event Free Survival Milestone Outcome (EFS-MO). Each list of differentially expressed genes is composed by the top 100 genes chosen according to different ranking methods, *i.e.*, *t*-test, SAM and LIMMA (preprocessed by the RMA method), and our approach (median *t*-value) which does not require a preprocessing algorithm. The average value of the overlap between the lists is calculated over 100 lists chosen randomly.

**Figure 4:**
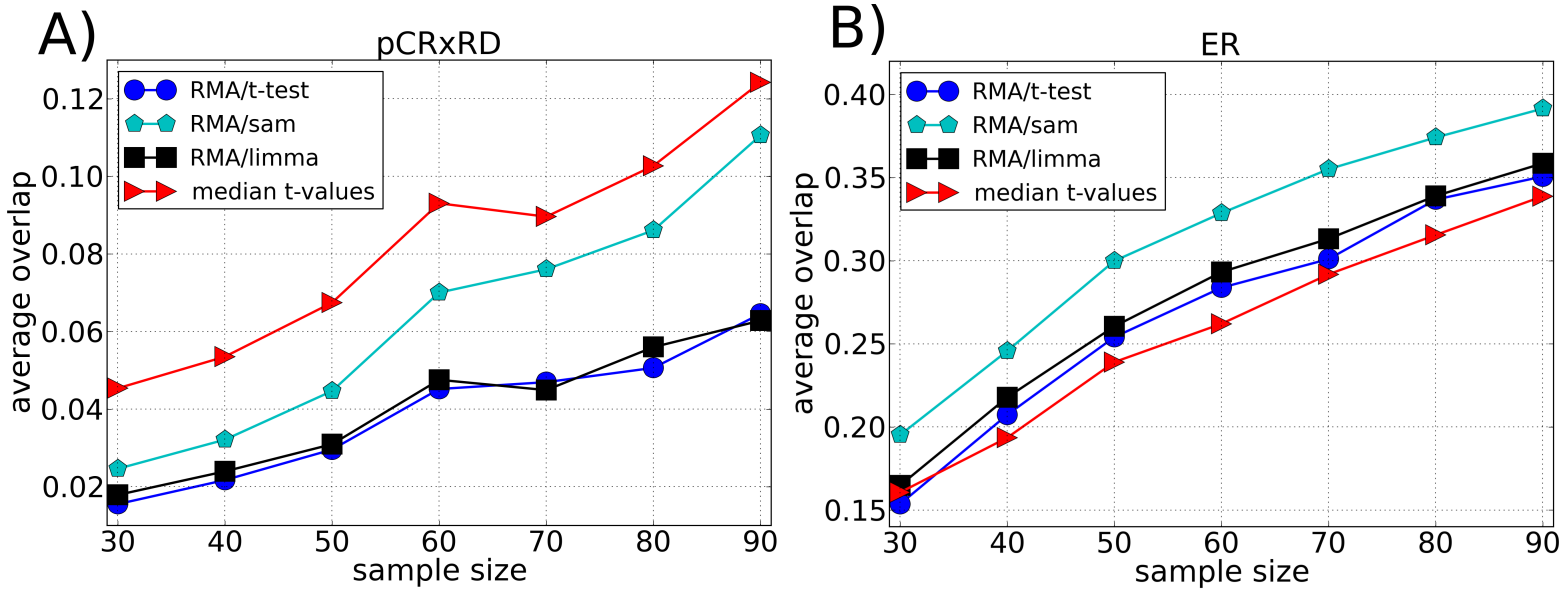
Average of the fraction of genes shared by two lists of differentially expressed genes (overlap) as a function of the sample size using the Breast Cancer dataset divided according to (**A**) pre-operative treatment response (pCR, pathologic complete response) and (**B**) estrogen receptor (ER) endpoint. Each list of differentially expressed genes is composed by the top 100 genes chosen according to different ranking methods, *i.e.*, *t*-test, SAM and LIMMA (preprocessed by the RMA method), and our approach (median *t*-value) which does not require a preprocessing algorithm. The average value of the overlap between the lists is calculated over 100 lists chosen randomly.

We compared the robustness of our methodology with different ranking methods: *t*-test, SAM and LIMMA, Figure 2, Figure 3 and Figure 4. The best performance of *t*-test, SAM and LIMMA was obtained using RMA as the preprocessing algorithm. Our approach (median *t*-value) shows a significantly higher overlap for the Leukemia and Multiple Myeloma datasets, Figure 2 and Figure 3, respectively. In the case of the Breast Cancer dataset, our approach shows a superior performance to the pre-operative treatment response (pCR, pathologic complete response) endpoint, but an inferior performance to the estrogen receptor (ER) endpoint, Figure 4. These results suggest that part of the lack of robustness in microarrays analysis is due to errors incorporated in the preprocessing steps, therefore explaining the significant gain of robustness of our methodology. However, we point out that for Breast Cancer and Multiple Myeloma, the levels of robustness for some analysis is still very low, suggesting a large biological heterogeneity among the samples.

## 4. Discussion

Over the years, a large number of preprocessing algorithms have been suggested. Many of them are based on underlying manipulations and assumptions that are difficult to understand. For example, the Plier method, suggested by Affymetrix, has been regarded as a good choice [28] despite being considered having biologically implausible assumptions [29]. Among the steps required for preprocessing, background correction is probably the most important since errors due to this step can more severely affect the genes with low expression values. Because of that, the standard deviation becomes dependent on the gene expression level. To overcome this issue, *t*-test modifications like SAM, LIMMA and other shrinkage methods have been developed and considered better choices. In fact, these methodologies present a good improvement in the task of identifying the differentially expressed genes when compared with standard *t*-test, as we showed using spike-in experiments. However, we highlight that by modifying the pooled variance, these methods tend to ignore the genes with low differences in expression and also, although improving the sensitivity when compared with standard *t*-test, these strategies do not show a significant improvement in the task of selecting a robust predictive list.

Here, we introduce an alternative approach for statistical analysis of microarray data that skips the background correction step, leading to a more powerful and robust test. Our procedure makes use of the standard *t*-test location- and scale-invariance property and relies on a well-established model that relates the probe intensity level with the gene expression level. Our method is easy to understand and to implement, however it does not offer an estimate of the expression level for each gene. We highlight that if the question under consideration is the identification of differentially expressed genes (DEG) or the predictive gene lists (PGLs), then intermediate estimation of expression levels is an unnecessary detour, and our method is useful. We also point out that our methodology is useful when the important genes are expected to have a small difference in expression. In this case, shrinkage methodologies are not recommended since they tend to ignore genes with small fold change.

## Appendices

**Table A1:** Latin Square design, which consists of 14 spike-in gene groups in 14 experimental groups with 3 repetitions.

gene/exp	1–3	4–6	7–9	10–12	13–15	16–18	19–21	22–24	25–27	28–30	31–33	34–36	37–39	40–42
1–3	0	0.125	0.25	0.5	1	2	4	8	16	32	64	128	256	512
4–6	0.125	0.25	0.5	1	2	4	8	16	32	64	128	256	512	0
7–9	0.25	0.5	1	2	4	8	16	32	64	128	256	512	0	0.125
10–12	0.5	1	2	4	8	16	32	64	128	256	512	0	0.125	0.25
13–15	1	2	4	8	16	32	64	128	256	512	0	0.125	0.25	0.5
16–18	2	4	8	16	32	64	128	256	512	0	0.125	0.25	0.5	1
19–21	4	8	16	32	64	128	256	512	0	0.125	0.25	0.5	1	2
22–24	8	16	32	64	128	256	512	0	0.125	0.25	0.5	1	2	4
25–27	16	32	64	128	256	512	0	0.125	0.25	0.5	1	2	4	8
28–30	32	64	128	256	512	0	0.125	0.25	0.5	1	2	4	8	16
31–33	64	128	256	512	0	0.125	0.25	0.5	1	2	4	8	16	32
34–36	128	256	512	0	0.125	0.25	0.5	1	2	4	8	16	32	64
37–39	256	512	0	0.125	0.25	0.5	1	2	4	8	16	32	64	128
40–42	512	0	0.125	0.25	0.5	1	2	4	8	16	32	64	128	256

**Table A2:** Class distribution of the datasets. The Breast Cancer and Multiple Myeloma datasets were obtained from the MAQC consortium [25]. The training and validation datasets were generated by different experimental groups. In our analysis we considered all samples: both training and validation samples.

Dataset	Training Set			Validation Set		
	Number of Samples	Positive	Negative	Number of Samples	Positive	Negative
Breast Cancer (pCR)	130	33	97	100	15	85
Breast Cancer (ER)	130	80	50	100	61	39
Multiple Myeloma (OS-MO)	340	51	289	214	27	187
Multiple Myeloma (EFS-MO)	340	84	256	214	34	180
